# Diabetes Prevalence and its Risk Factors in Rural Area of Tamil Nadu

**DOI:** 10.4103/0970-0218.69262

**Published:** 2010-07

**Authors:** Sanjay Kumar Gupta, Zile Singh, Anil J Purty, M Kar, DR Vedapriya, P Mahajan, J Cherian

**Affiliations:** Department of Community Medicine, Pondicherry Institute of Medical Sciences, Kalapet, Pondicherry - 605 014, India

**Keywords:** Diabetes mellitus, Indian diabetes risk score, rural area, risk factors, obesity

## Abstract

**Objective:**

To estimate the usefulness of the Indian diabetes risk score for detecting undiagnosed diabetes in the rural area of Tamil Nadu.

**Materials and Methods:**

The present study was conducted in the field practice area of rural health centers (Chunampett and Annechikuppam, Tamil Nadu), covering a population of 35000 from February to March 2008 by using a predesigned and pretested protocol to find out the prevalence and the risk of diabetes mellitus in general population by using Indian diabetes risk score.

**Results:**

1936 respondents comprising 1167 (60.27%) females and 769 (39.73%) males were studied. Majority 1203 (62.50%) were Hindus. 1220 (63.%) had studied up to higher secondary. 1200 (62%) belonged to lower and lower-middle socio-economic class. A large number of the subjects 948 (50%) were below 35 years of age. Most of the respondents 1411 (73%) indulged in mild to moderate physical activity. 1715 (87.91%) had no family history of diabetes mellitus. 750 (39.64%) individuals were in the overweight category (>25 BMI). Out of these overweight persons, 64% had high diabetic risk score. It is observed that chances of high diabetic score increase with the increase in BMI. Prevalence of diabetes in studied population was 5.99%; out of these, 56% known cases of diabetes mellitus had high (>60) IDRS. Co-relation between BMI and IDRS shows that, if BMI increases from less than 18.50 to more than 30, chances of high risk for developing diabetes mellitus also significantly increase.

**Conclusions:**

This study estimates the usefulness of simplified Indian diabetes risk score for identifying undiagnosed high risk diabetic subjects in India. This simplified diabetes risk score has categorized the risk factors based on their severity. Use of the IDRS can make mass screening for undiagnosed diabetes in India more cost effective.

## Introduction

Great efforts have been made by developed countries to control infectious diseases, but non-communicable diseases have not received much attention. Diabetes mellitus is one of the non-communicable diseases which have become a major global health problem. The International Diabetes Federation (IDF) estimated that there are 100 million people with diabetes worldwide that is about 6% of all adults.(1) This figure is expected to reach around 240 million by 2010.(1) In Asia, prevalence of diabetes is high and it has been estimated that 20% of the current global diabetic population resides in South- East Asia. Indeed, the number of cases in India is likely to double in two decades that is from 39.9 million (in 2007) to 69.9 million by 2025.(2,3) The study done by Indian Council of Medical Research (ICMR) in the year 1970 reported a prevalence of 2.3% in urban areas, which had increased to 12-19% in the year 2000. Correspondingly, in rural areas, prevalence rates had increased from 1% to 4-10%, and in the other study it was reported to be 13.2%.(4–6) Thus, it is clear that both in urban and rural India, prevalence rates of diabetes are increasing rapidly with estimation of 2:1 to 3:1. These prevalence rates are being maintained from the last 2-3 decades but in Kerala where rural prevalence rates are caught up or overtaken urban prevalence rates.(7–10)

The aim of this study is to assess the risk of diabetes mellitus in adults above 20 years, in rural area of Tamil Nadu using the Indian diabetes risk score (IDRS) developed by Mohan *et al*.(11)

1. The specific objectives are given as follows to study the prevalence of diabetes in rural population.
2. To estimate the usefulness of the Indian diabetes risk score (IDRS) for detecting undiagnosed diabetes in rural area.
3. To compare the prevalence of risk factors for diabetes among the known diabetic subjects and those without diabetes.

## Materials and Methods

This is a community-based cross-sectional (descriptive) study carried out in the field practice area of rural health centers (RHC) (Chunampett and Annachikuppam), Department of Community Medicine, Pondicherry Institute of Medical Sciences, Pondicherry. Covering the total population of 35,000, from these centers, four villages were selected by sampling (two from each centre), and all population above 20 years, presented on the day of survey and willing to participate were taken for the study. The total number of subjects surveyed from all four villages was 1936 (around 3% non-respondents) and duration of survey was from February to April 2008. In all the subjects, family history of diabetes was obtained and details on physical activities and other parameters were assessed, using a validated questionnaire by house-tohouse visit.(12) Waist measurements were obtained using a standardized technique. Socio-economic status was assessed according to modified BG Prasad classification based on CPI of April 2006,(13) and grade of physical activity was assessed by asking the following questions:

- How physical demanding is your work (occupation)?
- Do you exercise regularly in your leisure time?
- How would you grade your physical activity at home?

Than calculated the combined score of A+B+C = >3 vigorous-strenuous, 2 moderate, 1 mild, 0 sedentary. Analysis for high risk was done as per Indian diabetes risk score (IDRS) developed by Mohan *et al*. and parameters comprising two modifiable (waist circumference, physical activity) and two non-modifiable risk factors (age, family history) for diabetes. IDRS analysis was done with the help of all four parameters. If age <35 years score is = 0, if 35-49 years score is=20, if >50 years score= 30, waist circumference <80 cm for female and <90cm for male score = 0, >80-89 cm for female and >90-99 cm male score=10, >90 cm for female and >100 cm for male score=20, physical activities vigorous exercise or strenuous work score=0, moderate exercise work-home=10, mild exercise work/ home = 20, no exercise and sedentary work-home =30, family history of diabetes, no family history = 0, family history present either parent = 10, both parents =20. After adding all four parameters, if risk score (>60 very high risk, 30-50 moderate risk, <30 low risk). It is helpful to identify subjects at high risk for diabetes and also raised awareness about diabetes and its risk factors.

No ethical issues were involved as no intervention was carried out; however, verbal consent was obtained to proceed with the survey.

## Results

A total of 1936 respondents were interviewed. Among them, 774 (40%) were males and 1162 (60%) females. Majority 1200 (62%) were Hindus. 634 (32.80%) belonged to lower social class [Table 1]. Most of the respondents (90%) were non-vegetarian. Majority of males 496 (64.50%) had waist circumference of <90 cm. 288 (24.8%) females with high risk score had waist circumference of >90 cm which is significantly higher than males 68 (8.84%) with waist circumference of >100 cm. According to the physical activity, most of them 1411 (73%) belonged to mild to moderate category. Majority 1715 (88.58%) of the respondents had no family history of diabetes mellitus. 946 subjects (50%) were found in moderate risk (IDRS 30-50) for diabetes and 353(18.66%) had high risk for diabetes (IDRS>60) [Table 2]. Significant difference was observed between known cases of diabetes mellitus who had high (>60) IDRS (56%) than in general population (19%). It is observed that in underweight category (BMI<18.50) only 14.6% had high IDRS whereas in obese category (BMI >30) 40% had high IDRS and difference between these two groups were significantly high (*P*<0.05) [Table 3]. 750 (39.64%) are overweight (BMI >25). It was also observed, that out of these overweight individuals, (64%) had high diabetes risk score. The chances of high diabetes risk score are lower (14.61%) among individuals who are underweight (BMI<18.50) than those having BMI >30(40%) [Table 4]. The prevalence observed on the basis of known status of diabetes in rural community of Tamil Nadu was 116 (5.99%), out of that 65 (56%) had high IDRS [Table 4]. In the general population, prevalence of obesity (BMI>30) was 9.14%; it is almost double in known diabetic cases (17.24%). Prevalence of waist hip ratio >1 among males in general population was 3.56% and among females (>0.85) 32%. Difference between male and female waist hip ratio was significantly high (*P*<0.05).

**Table 1:** Distribution of respondents according to sociodemographic profile

Category	Number	Percentage
Age group (years)		
20-34	948	49
35-49	523	27
>50	465	24
Sex		
Male	774	40
Female	1162	60
Religion		
Hindu	1200	62
Muslim	600	31
Christian	136	7
Educational status		
Illiterate	484	25
Primary to middle	872	45
High school	348	18
Higher secondary	175	9
Graduate and above	58	3
Occupation		
Housewife	931	48.14
Laborers	292	15.08
Business	118	6.10
Agriculture	98	5.06
Retired	51	2.63
Student	44	2.27
Service	35	1.81
Others	366	19
Socio-economic status		
Upper class	111	5.73
Upper middle	244	12.60
Middle	385	20
Lower middle	562	20
Lower	634	32.80

**Table 2:** Distribution of respondent according to IDRS category

Score category	Number	Percentage
>60 (very high risk)	353	18.66
30-50 (Moderate risk)	946	50
<30 (Low risk)	593	31.34

Total	1892	100

**Table 3:** Distribution of respondent according to their body mass index (BMI) status and diabetes risk as per Indian diabetes risk score (IDRS)

Body mass index	Diabetes risk as per Indian diabetes risk score			
	Low risk	Moderate	Very high	Total
<18.50 (Underweight)	64 (37.42)	82 (47.95)	25 (14.61)	171 (100)*P*<0.05
18.5-24.99 (Normal range)	361 (37.17)	486 (50.05)	124 (12.77)	971 (100) *P*<0.05
25-29.99 (Pre-obese/Over weight)	149 (25.82)	239 (50.77)	135 (23.93)	577 (100) *P*>0.05
30 and above (Obese)	19 (10.98)	85 (49.13)	69 (39.88)	173 (100) *P*<0.05

Total	593 (31.34)	946 (50)	353 (18.66)	1892 (100)

Risk for diabetes significantly increases with the increase in BMI from normal to obese stage (*P*<0.05), Figures in parentheses are in percentage

**Table 4:** Distribution of respondents according to their known status of diabetes and IDRS

Known cases of diabetes (*N*=1936)		High IDRS in known diabetic (*N*=116)	
Number	Percentage	Number	Percentage
116	5.99	65	56

Prevalence of known cases of diabetes in studied population was 116 (5.99%), out of that large number 65 (56%) had high IDRS (>60) than general population (19%) which is statistically significant (*P*<0.05).

Similarly the prevalence of W/H ratio among known diabetics was also studied. It was observed that W/H ratio>1 in known cases of diabetes among males (50%) was significantly higher than general population (20%). Similarly known cases of diabetes among females with W/H ratio >0.85 were significantly higher (72%) than general population (25%).

## Discussion

In this study, we used simplified Indian diabetes risk score for identifying newly diagnosed high risk subjects in the rural Tamil Nadu. This is of great significance as use of such scoring system can prove to be a cost effective tool for screening of diabetes. Further use of such a risk score would be of great help in developing countries like India where there is a marked explosion of diabetes and over half of them remain undiagnosed. 19% of population had high risk score (>60) for diabetes [Table 2]. In a similar study conducted at Chennai by Mohan *et al*. 43% of the population were found in high risk category and another study done by us in urban area of Pondicherry had 31.2% high risk subjects. This risk difference may be due to variance in life-styles of the population as our study was done in a rural area, whereas Mohan *et al* conducted the study in a metropolitan city and our another study was in the urban area of Pondicherry. Prevalence of most risk factors was very high among known diabetics compared to people with IDRS >60, it retrospectively proved that if prevalence of risk factors is not reversed, one is likely to get diabetes.

Further confirmation with GTT is required among subjects with IDRS >60 to early detect the occurrence of diabetes. Besides this, lifestyle and dietary modification are to be initiated to reverse the risk factors among these groups.

Various studies in the west used different diabetes risk scores, based on simple anthropometric, demographic and behavioral factors, to detect undiagnosed diabetes.(14–17) We also used diabetes risk score suitable for detecting undiagnosed diabetes in South Asia. The risk score used in this study are those recommended by American Diabetes Association.(18) Compared to other studies IDRS has the following merits: its use is simple, scores are easily obtainable and have been drawn from high risk population. In addition the score is developed from representative sample of a large metropolitan city of India, the demographic of which is similar to rest of the India. According to the study “Urban rural differences in prevalence of self-reported diabetes in India,” people with sedentary lifestyle had diabetes.(19) In our study we also found that people with sedentary and mild physical activity had a higher risk for diabetes. According to the study conducted by Ramachandran *et al*. in an urban area of south India,(20) 47% of the people who had diabetes had a positive family history and the other study competed by us in urban area of Pondicherry had 31.50% positive family history while in the present study only 12% of the respondents gave a positive family history. This difference may be due to different life-styles and socio-economic status of the respondents.

## Conclusion

This study estimates the usefulness of simplified Indian diabetes risk score for identifying high risk diabetic subjects in the community. This simplified diabetes risk score has categorized the risk factors based on their severity. Use of the IDRS can make mass screening for diabetes in India more cost effective.
